# Biodiversity, Distribution and Functional Differences of Fungi in Four Species of Corals from the South China Sea, Elucidated by High-Throughput Sequencing Technology

**DOI:** 10.3390/jof10070452

**Published:** 2024-06-27

**Authors:** Wenyu Dong, Jiatao Chen, Xinyu Liao, Xinye Chen, Liyu Huang, Jiayu Huang, Riming Huang, Saiyi Zhong, Xiaoyong Zhang

**Affiliations:** 1 University Joint Laboratory of Guangdong Province, Hong Kong and Macao Region on Marine Bioresource Conservation and Exploitation, College of Marine Sciences, South China Agricultural University, Guangzhou 510642, China; idwy1207@outlook.com (W.D.); 15889168350@163.com (L.H.); huangjiayu@scauaie.cn (J.H.); 2 Guangdong Provincial Key Laboratory of Aquatic Product Processing and Safety, College of Food Science and Technology, Guangdong Ocean University, Zhanjiang 524088, China; 3 Guangdong Provincial Key Laboratory of Nutraceuticals and Functional Foods, College of Food Science, South China Agricultural University, Guangzhou 510642, China; huangriming@scau.edu.cn

**Keywords:** high-throughput sequencing, coral, fungal diversity

## Abstract

Recent studies have predominantly spotlighted bacterial diversity within coral microbiomes, leaving coral-associated fungi in the shadows of scientific inquiry. This study endeavors to fill this knowledge gap by delving into the biodiversity, distribution and functional differences of fungi associated with soft corals *Cladiella krempfi* and *Sarcophyton tortuosum*, gorgonian coral *Dichotella gemmacea* and stony coral *Favia speciosa* from the South China Sea. Leveraging high-throughput sequencing of fungal internal transcribed spacer-1 (ITS1) region of the rRNA gene, a total of 431 fungal amplicon sequence variants (ASVs) were identified in this study, which indicated that a large number of fungal communities were harbored in the South China Sea corals. Noteworthy among our findings is that 10 fungal genera are reported for the first time in corals, with *Candolleomyces*, *Exophiala*, *Fomitopsis*, *Inaequalispora*, *Kneiffiella*, *Paraphaeosphaeria*, and *Yamadazyma* belonging to the Ascomycota, and *Cystobasidium, Psathyrella*, and *Solicoccozyma* to the Basidiomycota. Moreover, significant differences (*p* < 0.05) of fungal communities were observed among the various coral species. In particular, the gorgonian coral *D. gemmacea* emerged as a veritable haven for fungal diversity, boasting 307 unique ASVs. Contrastingly, soft corals *S. tortuosum* and *C. krempfi* exhibited modest fungal diversity, with 36 and 21 unique ASVs, respectively, while the stony coral *F. speciosa* hosted a comparatively sparse fungal community, with merely 10 unique ASVs in total. These findings not only provide basic data on fungal diversity and function in the South China Sea corals, but also underscore the imperative of nuanced conservation and management strategies for coral reef ecosystems worldwide.

## 1. Introduction

As unique ecosystems within the ocean realm, coral reefs are celebrated for their extraordinary biodiversity and substantial primary productivity, playing a pivotal role in sustaining the ocean’s ecological equilibrium [1,2]. Not only do they serve as bastions of biological diversity, but they also offer invaluable resources such as food, medicine, and construction materials, underpinning various aspects of human livelihoods [3]. Beyond their economic value, coral reefs significantly contribute to environmental protection and coastal flood defense [4], with their structural complexity being foundational to their ecological functions [5]. This structural intricacy not only furnishes diverse habitats for marine organisms, fostering species growth and biodiversity [6], but is also closely associated with the density and biomass of fish, crucial for upholding the stability of marine food chains [7]. Particularly in the South China Sea, the value of coral reefs is manifested not only in their ecological functions but also in their economic potential. Economically, the annual value of coral reefs in the South China Sea has been estimated at approximately CNY 15.65 billion, with the potential to increase to CNY 137 billion over 20 years, underlining the importance of sustainable development for their future value [8].

However, coral reefs are currently under unprecedented threats from anthropogenic activities like coastal development and pollution, alongside the impacts of global climate changes, such as rising sea temperatures and levels, causing widespread degradation and bleaching events [9]. An increase in coral diseases, often linked to the imbalance of surface-associated microbes, exacerbates the decline in coral populations [10]. Contrary to the previous perceptions of their uniformity and stability, coral reefs are now recognized to be heterogeneous, delicate, and globally pressured ecosystems [11]. The health and resilience of coral reefs significantly depends on their interaction with microbial communities, which are foundational for ecosystem stability and functionality, as well as crucial determinants of coral adaptability to environmental perturbations [12].

Symbiotic microbes associated with corals mainly include bacteria, fungi and microalgae, which are indispensable for maintaining coral health. Among them, fungi play a significant role, particularly in nutrient cycling and protecting corals from pathogens through the production of antibiotics and other bioactive compounds [13,14]. Their involvements in processes such as nitrogen fixation, sulfur compound metabolism, and quorum sensing form a protective barrier against external pathogens [15]. Changes in microbial compositions are associated with disease and bleaching, underlining the connection between microbial communities and coral health [16]. Exploring the interactions between microbial communities and coral immune systems may uncover ancient, evolutionarily conserved mechanisms crucial for determining the outcomes of host–microbe interactions and influencing the host’s resilience [17]. Moreover, the rapid adaptability of microbial communities to environmental changes enables corals to withstand climate changes and other environmental pressures [18]. During corals’ lifecycle, symbiotic microbes also aid in the recruitment and settlement of larvae, bolstering coral reefs’ resilience [19]. Thus, microbial communities play a fundamental role in ensuring corals’ physiological balance and environmental adaptability, essential for the long-term health of coral reefs and ecosystem stability [20].

Recent studies on fungi in corals have garnered increasing attention within the field of coral-associated microbes [21]. Studies on isolating fungi from various coral species across different regions have unveiled a considerable diversity within the fungal kingdom, encompassing phyla such as Ascomycota, Basidiomycota, and Chytridiomycota [22,23]. Particularly, investigations into cultivable coral fungi have confirmed the presence of diverse fungi in the South China Sea corals, including genera like *Aspergillus* and *Penicillium* [24,25,26]. These findings not only highlight the diversity of fungi associated with corals but also underline the variability in fungal distribution across coral species. High-throughput sequencing technologies have further facilitated in-depth analyses of uncultivable coral-associated fungi, identifying 19 symbiotic fungi with antibacterial properties from the South China Sea corals, underscoring their potential contributions to coral health and disease prevention [27]. Such discoveries have significantly stimulated interest in coral symbiotic fungi research, offering fresh insights into the pivotal role of fungi in coral ecosystems and establishing a foundation for the exploration of novel bioactive compounds.

Research on coral-associated fungi has traditionally relied on microscopic observation and culture techniques [28,29]. Microscopic observation involves examining coral tissue sections under a microscope to preliminarily identify and classify fungi, though it is limited by the morphological similarity of fungi, making accurate distinction difficult. Culture techniques involve growing fungi from coral tissues in the laboratory to study their physiological characteristics and ecological functions, but these methods typically only detect fungi that can grow on artificial media, overlooking numerous unculturable fungi. Traditional studies also include environmental sample DNA sequencing to identify fungal species associated with corals. Despite providing some DNA sequence information, these culture-dependent methods reveal limited fungal diversity [30]. Recently, high-throughput sequencing (NGS) has expanded our understanding of coral-associated fungal diversity. NGS allows comprehensive sequencing of fungal DNA in coral tissue samples, revealing the complete microbial community structure, including uncultivable cryptic fungi, and addressing the limitations of traditional methods [31]. This technology has elucidated the complex symbiotic relationships between corals and various unknown or unclassified fungi, uncovering microbes potentially unviable under standard laboratory conditions, thus enriching our comprehension of coral ecosystem complexity and environmental adaptability.

Despite growing scientific interest in the diversity and secondary metabolites of coral symbiotic fungi, the exploration of fungal biodiversity within corals remains inadequate [32]. Many studies rely on culturing methods that may not capture the full spectrum of fungal diversity, potentially leading to underestimation. Additionally, research into the functional roles and symbiotic mechanisms of coral-associated fungi is scarce. Leveraging high-throughput sequencing technology, this study aims to comprehensively explore fungal diversity and distribution patterns in four species of the South China Sea corals. Our findings will enhance the understanding of fungal diversity within these corals and provide a vital data foundation for future research on coral pathology, essential for predicting and mitigating coral diseases and offering novel insights and strategies for coral ecosystem conservation and sustainable utilization.

## 2. Materials and Methods

### 2.1. Sample Collection

**Figure 1:**
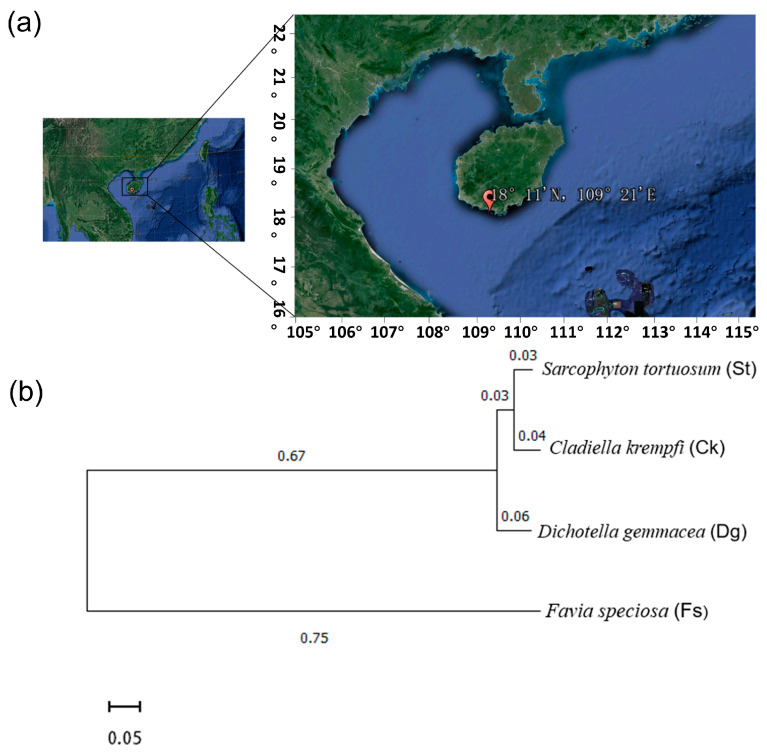
Map of the South China Sea and the location of the coral sampling site (**a**), and Neighbor joining phylogenetic tree (**b**) based on the cytochrome c oxidase subunit I (COI) gene of four corals from the South China Sea, constructed using the maximum composite likelihood method and a bootstrap method with 1000 replicates, with mega 6.0 software. The clone sequences of the coral samples were obtained from NCBI with the locus tags JX991253.1, JX991224.1, KF955068.1, AB441 194.1. The map is sourced from Google Maps.

In August 2021, samples of the South China soft corals *Sarcophyton tortuosum* (*St*) and *Cladiella krempfi* (*Ck*), gorgonian coral *Dichotella gemmacea* (*Dg*), and stony coral *Favia speciosa* (*Fs*) were obtained from the pristine environs of the Sanya Coral Reef Nature Reserve, Hainan (18°11′ N, 109°25′ E) (Figure 1). Three biological replicates were collected from each coral species. These coral samples were dispersed over an area of approximately two square kilometers within the Sanya Coral Reef Nature Reserve in order to account for small-scale spatial differences in fungal communities and avoid sampling of coral clone mates. Following each coral species’ swift retrieval, they were swiftly placed in a sterile sealed bag and promptly nestled within ice-filled containers for preservation during transport to the laboratory. Upon arrival, the specimens were expediently stored at −80 °C, ensuring the preservation of their biological integrity, preparatory to the subsequent DNA extraction.

Initially preserved, the coral samples collected underwent genetic analyses to elucidate their evolutionary relationships, crucial for revealing the genetic diversity. The utility of cytochrome c oxidase subunit I (COI) gene sequences for species differentiation among corals had been well-established. Consequently, a phylogenetic tree, informed by COI gene sequences sourced from the National Center for Biotechnology Information (NCBI, Bethesda, MD, USA), was constructed to depict the evolutionary linkages across four coral species (Figure 1). This tree illustrated a significant kinship between corals *St* and *Ck*, as evidenced by a robust bootstrap value. Conversely, corals *Dg* and *Fs* exhibited a more remote connection, denoted by lesser bootstrap values and extended branch lengths, highlighting their distinct genetic backgrounds.

### 2.2. DNA Extraction and High-Throughput Sequencing

The coral samples were first rinsed in 75% ethanol solution for 30 s to remove surface contaminants, and then washed thrice with sterile seawater to eliminate surface microbes and mucus. Subsequent homogenization occurred in a sterile mortar with a liquid nitrogen-cooled pestle. Approximately 0.5 mL of the resulting homogenate was preserved in 0.8 mL TE buffer for DNA extraction. The DNA was extracted using the E.Z.N.A. Soil DNA Kit (Omega Bio-Tek, Norcross, GA, USA), and its integrity confirmed via agarose gel electrophoresis.

The fungal internal transcribed spacer-1 (ITS1) region of ribosomal RNA gene was selected for the taxonomic analysis in this study because it has higher sequence variability than the ITS 2 region, ITS1 is more widely used to study fungal diversity, and a higher amount of sequences for this region can be found in databases [33]. The ITS rDNA was amplified using ITS1 and ITS2 primers, selected for their fungal specificity and breadth, thus enabling comprehensive fungal diversity detection within the coral species. PCR optimization—based on annealing temperatures, extension times, and cycle numbers—followed protocols established by Xie et al. [34]. The optimized thermal cycling conditions included an initial denaturation at 94 °C for 5 min, followed by 31 cycles (94 °C for 30 s, 53 °C for 30 s, 72 °C for 45 s) and a final extension at 72 °C for 10 min. The PCR mixture, totaling 60 μL, comprised 30 μL PhusionR High-Fidelity PCR Master Mix (New England Biolabs, Ipswich, MA, USA), 0.4 μM of each primer, and approximately 20 ng of DNA template. Post-amplification, PCR products were purified with the GeneJET Gel Extraction Kit (Thermo Scientific, Vantaa, Finland).

The ITS rDNA sequencing library was then constructed using the NEB Next DNA Sample Prep Kit, according to the manufacturer’s instructions, and sequenced on the Illumina MiSeq platform (Version 3), ensuring each library was duly indexed.

To ensure comparability across different samples, we standardized the results by normalizing the read counts of each sample to account for variations in sequencing depth. This standardization process involved scaling the read counts to the median sequencing depth across all samples, thus allowing for accurate comparisons of fungal community composition and relative abundances. The details of this standardization process are reflected in the footnote of Table 1.

### 2.3. Sequence Analysis

Raw paired-end sequences from each sample, acquired via the Illumina platform, were processed using the QIIME2-2023.2 platform for detailed analysis [35]. Initially, sequences were segregated based on DNA barcodes, followed by the removal of primers and low-quality reads via the QIIME2 demux command. The DADA2 plugin facilitated sequence quality control by trimming low-quality reads and those with ambiguous bases. Subsequently, forward and reverse reads that passed quality checks were merged and allocated to their respective samples using DNA barcodes. The UCHIME plugin identified and eliminated chimeric sequences. Amplicon sequence variants (ASVs) were then defined by clustering effective sequences at a 97% similarity cutoff using the VSEARCH plugin, with each ASV represented by its most abundant sequence.

### 2.4. Diversity Analysis

Subsequent to processing in QIIME2, the biom-format data were converted into a text file for analysis of fungal community diversity. This conversion facilitated compatibility with additional analytical software. Diversity metrics, including rarefaction curves, α-diversity (Good’s coverage, Chao1 richness, Richness index, Shannon, and Simpson diversity indices), and β-diversity (principal coordinate analysis, PCoA), were computed at the ASV level using the Bray–Curtis index. Analysis utilized the “GUnifrac”, “ggplot2”, and “vegan” packages within Rstudio [36,37]. The Bray–Curtis index, employed for its ability to reflect species’ relative abundances without considering evolutionary relationships, served as the basis for measuring fungal community similarities. Interspecific comparisons were conducted using the “vegan” and “ggplot2” packages in Rstudio [36]. Further visualization and analysis of fungal diversity were performed with the “pheatmap”, “ggtern”, “ggpub”, and “circlize” packages in Rstudio [38,39,40].

### 2.5. Taxonomic and Functional Classification

Utilizing the q2-feature-classifier plugin, the classifier was calibrated with the UNITE QIIME for Fungi Version 16.10.2022 database, which was specifically designed for fungal taxonomy and contains over 1 million annotated, quality-controlled fungal sequences [41]. The Naive Bayes method was employed for sequence classification, and the sequences not assignable to the genus level through this method were further scrutinized using unite online blast and Genbank blast for more precise identification [42]. A phylogenetic tree was then constructed with the q2-phylogeny plugin, employing the maximum likelihood method, which accounts for the evolutionary model and sequence confidence.

In a Python 3 environment, the FUNGuild script was utilized to predict fungal functional groups [43]. This tool categorizes fungi into various functional groups based on criteria such as ecological niche, nutritional mode, and host range, including lignin decomposers, pathogens, endophytes and others.

### 2.6. Nucleotide Sequence Accession Number

These ITS-rDNA sequences corresponding to all the coral samples have been submitted to the NCBI Sequence Read Archive. The sequences are readily accessible under the accession number PRJNA1101115.

## 3. Results

### 3.1. High-Throughput Sequencing and Sequence Analysis

**Figure 2:**
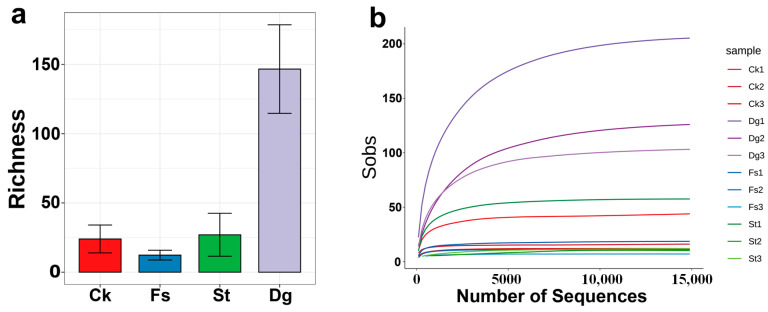
Comparative fungal ITS sequence richness (**a**) and rarefaction curves (**b**) in coral samples from the South China Sea. The bar graph represents the mean species richness of fungal ITS sequences among four coral species, including gorgonian coral *Dichotella gemmacea* (*Dg*), and soft corals *Cladiella krempfi* and *Sarcophyton tortuosum* (*Ck* and *St*), as well as stony coral *Favia speciosa* (*Fs*). Error bars denote the standard deviation, indicating the variability in fungal richness across samples.

The fungi of four coral species from the South China Sea, including gorgonian coral *Dg*, soft corals *Ck* and *St*, and stony coral *Fs*, were studied using high-throughput ITS-rRNA gene sequencing. A total of 390,013 fungal ITS1 sequences were retrieved, grouped into 431 ASVs at a 97% similarity threshold. A comparison between the Chao1 index and observed Richness values at the 97% similarity level confirmed this representation (Table 1, Figure 2a). The rarefaction curves for corals *Dg*, *Ck*, *St* and *Fs* were plateaued, suggesting comprehensive capture of each coral’s fungal diversity (Figure 2b). Diversity indices were calculated based on ITS1 sequences of fungi retrieved from high-throughput sequencing of coral samples.

### 3.2. Fungal Diversity and Composition

**Figure 3:**
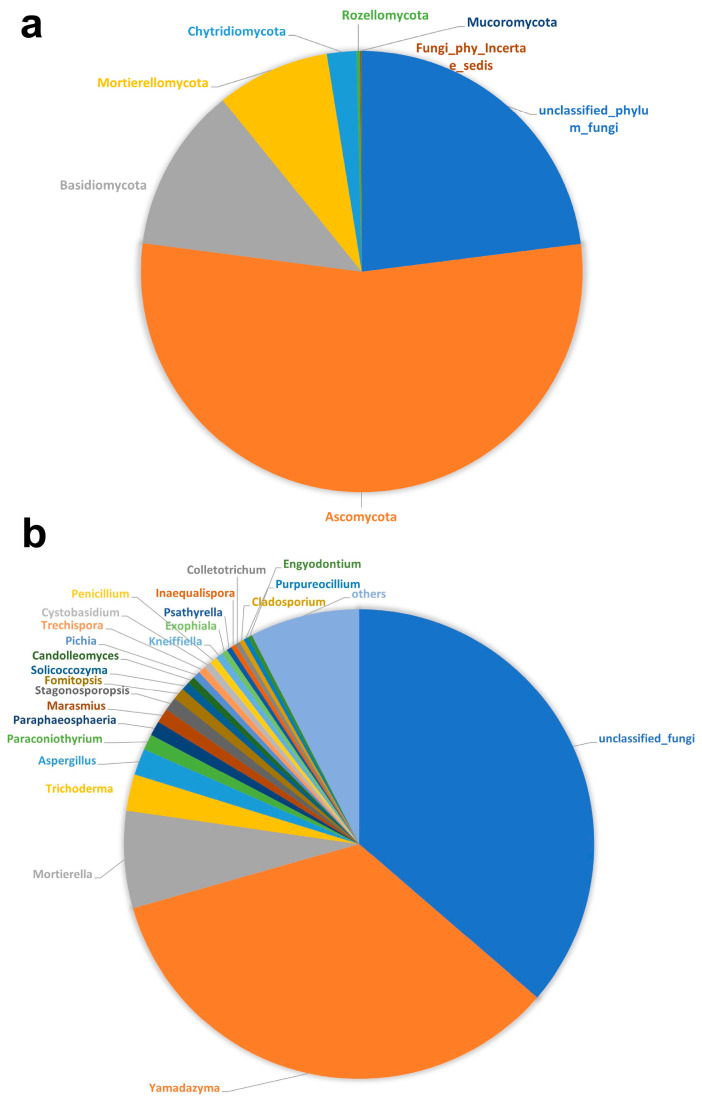
Coral-associated fungal community composition and relative abundance at phylum (**a**) and genus (**b**) level. The taxonomic examination identified 431 amplicon sequence variants (ASVs) across eight fungal phyla.

An extensive taxonomic examination revealed 431 ASVs across eight fungal phyla, including Ascomycota, Basidiomycota, Chytridiomycota, Mortierellomycota, Mucoromycota, Rozellomycota, and unclassified fungi, which encompassed both those unable to be placed within any known phylum (Fungi incertae sedis) and newly identified fungi pending classification. Furthermore, this sentence must be modified in the following sense. Among them, Ascomycota (54.07%) was the predominant phylum, followed by Basidiomycota (12.10%) and Mortierellomycota (8.30%), but with a considerably lower percentage of relative abundance (Figure 3a). A total of 179 fungal genera in 126 families were detected in these corals, and Corals *Ck*, *St*, *Dg* and *Fs* harbored 28 genera in 26 families, 34 genera in 33 families, 159 genera in 114 families and 15 genera in 14 families, respectively.

At the genus level, *Yamadazyma* (in Ascomycota) was most prevalent (34.25%), followed by *Mortierella* (in Mortierellomycota) (6.70%), *Trichoderma* (in Ascomycota) (2.50%), and *Aspergillus* (in Ascomycota) (1.85%). Those genera were followed by *Paraconiothyrium* (Ascomycota), *Paraphaeosphaeria* (Ascomycota), *Marasmiu* (*Basidiomycota*), and *Stagonosporopsis* (Ascomycota), which showed a relative abundance of 1.08%, 1.02%, 1.00% and 0.94%, respectively (Figure 3b). Noteworthy among our findings is that 10 fungal genera are reported for the first time in corals in this study. These newly identified genera were *Candolleomyces*, *Exophiala*, *Fomitopsis*, *Inaequalispora*, *Kneiffiella*, *Paraphaeosphaeria*, and *Yamadazyma* belonging to the Ascomycota, and *Cystobasidium, Psathyrella*, and *Solicoccozyma* to the Basidiomycota [44,45,46,47,48,49,50].

Additionally, the top 20 fungal genera within corals were quantified, revealing significant diversity with *Mortierella* fungi notably represented; while fungal genera like *Trichoderma*, *Aspergillus* and *Stagonosporopsis* were relatively abundant, their crucial roles in ecological balance underscored their ecosystem significance (Figure 3b).

### 3.3. Comparison of Fungal Communities in Different Species of Corals

**Figure 4:**
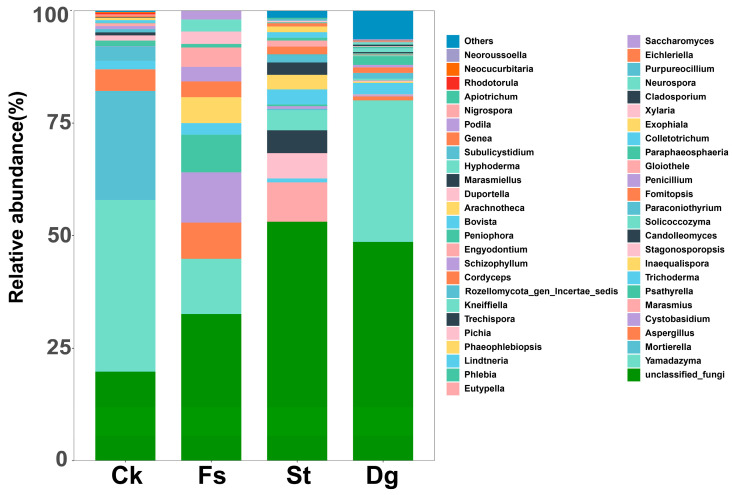
Fungal community composition of four coral species at the genus level. This graph displays the relative abundance distribution of fungal genera in four coral species from the South China Sea, including gorgonian coral *Dichotella gemmacea* (*Dg*), and soft corals *Cladiella krempfi* and *Sarcophyton tortuosum* (*Ck* and *St*), as well as stony coral *Favia speciosa* (*Fs*). Each bar corresponds to a different coral species, with color layers representing the percentage of each fungal genus’s relative abundance.

**Figure 5:**
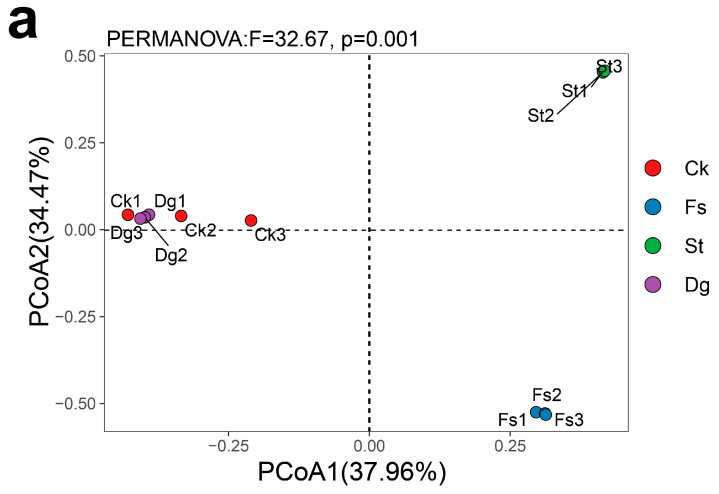

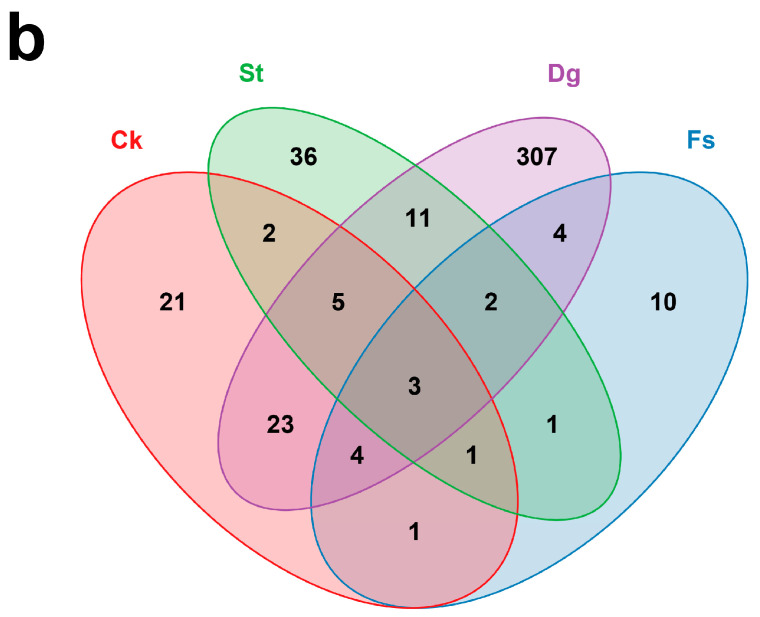
The PCoA plot (**a**) and Venn diagram (**b**) display the distinct fungal community structures among four coral species from the South China Sea, including gorgonian coral *Dichotella gemmacea* (*Dg*), and soft corals *Cladiella krempfi* and *Sarcophyton tortuosum* (*Ck* and *St*), as well as stony coral *Favia speciosa* (*Fs*). PCoA1 and PCoA2 axes explain 37.96% and 34.47% of the variance, respectively. Significant differences in fungal community structures were confirmed by PERM ANOVA analysis (F = 367, *p* = 0.001), indicating a clear segregation of the fungal communities across the coral species.

This research elucidated the diversity within fungal communities in four coral species, employing Shannon and Simpson indices for comprehensive diversity assessment and generating species richness curves (Figure 2). It was observed that coral *Dg* showcased the most considerable species richness, which was significantly higher than that of the other corals, indicating a robust and varied fungal association. In contrast, coral *Fs* exhibited the least species richness, while coral *Ck* demonstrated moderate richness, positioning itself between the high diversity of coral *St* and the lower diversity of coral *Fs*. Interestingly, despite coral *Dg*’s superior species richness, coral *St* demonstrated a higher diversity at the genus level (Figure 4), further confirming the complex nature of its fungal community. Each coral species was found to host a distinct fungal community or “specific microbiota”, with corals *Dg*, *Ck*, *St*, and *Fs* harboring 307, 21, 36 and 10 unique fungal ASVs, respectively (Figure 5b). Notably, a “core microbiota”, comprising three fungal ASVs found across all coral species, was exclusively attributed to unclassified fungi. For detailed information on the species composition and abundance data, please refer to the Supplementary Material (Table S1), which provides an in-depth analysis of the fungal communities associated with each coral species.

The results of PCoA further dissected the diversity among different coral species (Figure 5a), revealing significant variances (*p* < 0.05). The study uncovered 23 fungal ASVs shared between corals *Dg* and *Ck*, 11 between corals *Dg* and *St*, only one between corals *St* and *Fs*, and similarly, one between corals *Ck* and *Fs*, with coral *Dg* and coral *Fs* sharing four fungal ASVs (Figure 5b). The analysis extended to the richness and composition across different coral species, unraveling that the gorgonian coral *Dg* possessed the highest species richness, followed by the soft corals *St* and *Ck*, which exhibited comparably higher richness. In contrast, the stony coral *Fs* displayed the lowest species richness. Despite belonging to the same South Sea soft coral order, corals *Ck* and *St* showed significant differences in their genus-level species composition. For instance, genera such as *Mortierella*, *Yamadazyma* and *Pichia* were exclusive to coral *Ck*, whereas *Phaeophlebiopsis*, *Lindtneria*, and *Marasmius* were unique to coral *St*.

Among the top 20 fungal genera identified, *Yamadazyma* was prominent, notably within the fungal communities in coral *Ck*. Fungi in coral *Fs* were characterized by the prevalence of both *Yamadazyma* and *Cystobasidium*. Meanwhile, the fungal communities in coral *St* were distinguished by a high abundance of *Marasmius*. Additionally, *Paraconiothyrium* was more abundant than *Stagonosporopsis* in the overall fungal community composition. Additionally, coral *Dg* also exhibited a significant proportion of *Yamadazyma*.

### 3.4. Prediction of Fungal Functional Groups

**Figure 6:**
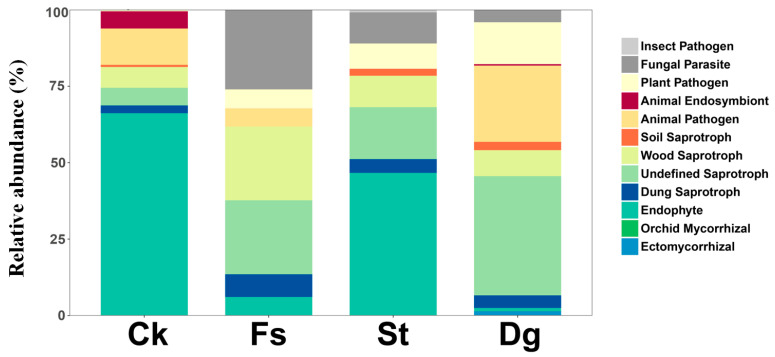
Relative abundance of fungal functional groups in coral species as predicted by FUNGuild. The bar chart displays the proportion of different fungal trophic modes within four coral species from the South China Sea, including gorgonian coral *Dichotella gemmacea* (*Dg*), and soft corals *Cladiella krempfi* and *Sarcophyton tortuosum* (*Ck* and *St*), as well as stony coral *Favia speciosa* (*Fs*).

Utilizing the FUNGuild database enabled the prediction of trophic modes of fungal communities at the genus level, categorizing them into groups such as insect pathogens, fungal parasites, plant pathogens, and various forms of saprotrophic fungi [43]. The analysis showed that undefined saprotrophic fungi were predominant in coral *Dg*; endophytes were the main groups in corals *Ck* and *St*; and coral *Fs* featured a mix of fungal parasites, wood-decaying fungi and undefined saprotrophic fungi (Figure 6). A further integration of diversity and composition analysis revealed a broad range of fungal genera shared between corals *Dg* and *Ck*, with pathotrophic-symbiotrophic genera absent in coral *St* and pathotrophic genera missing in coral *Ck*. Notably, coral *Dg* harbored 307 unique fungal genera, predominantly ectomycorrhizal. These ASVs included both classified and unclassified taxa, indicating a diverse range of fungal associations.

Quantitative analysis further indicated that endophytes constituted 35–45% of the coral *Ck* and 20–30% of coral *St*, while coral *Fs* exhibited fungal parasites, wood-decaying fungi, and undefined saprotrophic fungi with relative abundances in the ranges of 15–25%, 10–20%, and 30–40%, respectively. Coral *Dg* was characterized by a 40–50% abundance of undefined saprotrophic fungi and contained 307 unique genera, predominantly ectomycorrhizal. These findings provide a detailed overview of the fungal community composition across the sampled environments, laying a foundation for further ecological and biogeographical studies. The significant ecological diversity observed across samples may be influenced by host characteristics, environmental conditions, or yet-to-be-measured biogeographical factors.

## 4. Discussion

Impressively, a total of 431 ASVs were predominantly classified to eight fungal phyla, among which Ascomycota and Basidiomycota emerged as the most abundant, accounting for 54.07% and 12.10% of the total sequences, respectively. Additionally, our study is the first to report 10 fungal genera belonging to Ascomycota and Basidiomycota in corals, thereby reinforcing the significance of Ascomycota and Basidiomycota within coral fungal communities [51,52]. At the genus level, *Yamadazyma* emerged as the most prevalent, followed by *Mortierella*, *Trichoderma*, and *Aspergillus*, which is consistent with other results from the literature [53,54,55]. The detection of *Mortierella* across both terrestrial and marine sedimentary environments [56], alongside *Trichoderma* and the ubiquitous distribution of *Aspergillus* [57], suggests their potential contributions to ecological dynamics and interactions within coral ecosystems. However, these functional predictions should be considered preliminary, as they are based on database assignments and require further experimental validation. The capacity of *Mortierella* to augment nutrient cycling and organic matter decomposition suggested a parallel function in coral sediments, possibly affecting coral metabolism via nutrient availability [58]. Similarly, the established roles of *Trichoderma* in enhancing plant growth and resilience by bolstering stress tolerance may find analogous applications in coral environments, potentially modulating responses to environmental stress [59]. Furthermore, *Aspergillus*, renowned for its diverse enzymes and metabolites, could influence the bioavailability of nutrients or participate in detoxification processes within coral systems [60]. While direct associations between these fungi and specific functions in coral ecosystems are still in need of further verification, their recognized ecological roles elsewhere provide a foundation for hypothesizing their impact on coral metabolism and adaptability. This highlights the necessity of targeted functional studies to confirm these roles. Continued explorations of the precise interactions and mechanisms could reveal the extent of these fungi’s influence on coral health and resilience. The ecological functions of *Yamadazyma* fungi in coral ecosystems, although not yet definitively confirmed, hint at roles in organic matter decomposition and potentially calcification, given their capabilities in other ecosystems [61,62]. Additionally, their roles in nutrient cycling and pathogen defense on plant leaf surfaces may suggest symbiotic relationships within coral ecosystems, necessitating further investigation [63,64].

Notably, 10 fungal genera are reported for the first time in corals in this study, including *Candolleomyces*, *Cystobasidium*, *Exophiala*, *Fomitopsis*, *Inaequalispora*, *Kneiffiella*, *Paraphaeosphaeria*, *Psathyrella*, *Solicoccozyma* and *Yamadazyma*. Among them, the presence of *Exophiala* in deep-sea sediments underlines its distinct contribution to coral adaptation in harsh environments [65]. The identification of genera such as *Fomitopsis* and *Solicoccozyma*, traditionally associated with organic decomposition and nutrient cycling, hints at their potential roles within coral reef ecosystems—vital for maintaining ecological health [66,67]. Moreover, *Cystobasidium*’s symbiotic qualities further emphasize the complex interactions among fungi within coral ecosystems, essential for unraveling the intricacies of coral health and ecological resilience [68]. The discovery of these genera opens new avenues for understanding their ecological functions and interactions within coral ecosystems. For instance, *Yamadazyma* and *Candolleomyces* might significantly modulate microbial communities, potentially affecting coral health and disease resistance, similarly to the functions of related yeasts in diverse ecosystems [69,70]. The capabilities of *Paraphaeosphaeria* and *Kneiffiella* in organic decomposition or nutrient cycling within coral reefs point to fundamental processes pivotal for ecosystem vitality and warrant further exploration [71,72]. Although these findings shed light on the potential ecological impacts of these newly reported fungal genera in corals, detailed investigations are essential to delineate their precise roles within coral ecosystems. These insights are crucial for advancing our understanding of coral–microbial interactions, informing conservation strategies, and managing coral reef health more effectively.

Furthermore, this study lays a foundational basis for further taxonomic and functional investigations of coral-associated fungal communities, essential for advancing coral ecology and conservation biology. Significant variations in fungal diversity across different coral species notably highlight the differences in coral adaptability and resilience. By exploring the complex interactions between corals and fungi and integrating the existing literature, this research has unveiled their intricacies. The symbiotic relationship between corals and *Symbiodinium* (zooxanthellae) is crucial for coral health. These intracellular dinoflagellates provide essential nutrients through photosynthesis, enhancing the corals’ energy budget and promoting calcification [73]. *Symbiodinium* is phylogenetically diverse, with various clades showing different ecological preferences and thermal tolerances, influencing coral resilience [74]. The distribution of *Symbiodinium* varies among coral species, with some hosting multiple clades that can shift in response to environmental stressors [75]. This dynamic relationship suggests that interactions between fungal communities and *Symbiodinium* could affect coral health and stress responses. Although this study focuses on fungal diversity, further investigation into the relationships between fungi and *Symbiodinium* is warranted. Broader sampling of coral species and their microbial communities is essential to understand these interactions and their implications for coral resilience under changing conditions.

Interpreting these complex interactions is crucial for understanding the ecological roles of fungi in coral health. The notable fungal diversity observed in coral *Dg* may suggest ecological adaptability [76]. Our findings also hint at fungal diversity’s role in bolstering coral disease resistance, aligning with the results of Thurber et al. [77]. Conversely, coral *St*, despite its lower fungal diversity, exhibits a richer species composition, possibly as an adaptive response to environmental selection pressures. This diversity, critical for ecosystem functionality as Carturan et al. noted [78], contrasts with the minimal diversity in corals *Ck* and *Fs*. Their specificity might stem from reliance on certain environmental factors and heightened sensitivity to changes, suggesting a specialized ecological niche adaptation. Our analysis, supported by Crowther et al. [79], indicates that corals selectively associate with certain fungal populations, reflecting niche adaptation and survival tactics within their respective ecological groups. Such specificity in host–microbe relationships may confer adaptive benefits to corals [80]. Moreover, this study highlights the uniqueness of fungal communities (corals *Dg*, *Ck*, *St*, and *Fs* harbored 307, 21, 36, and 10 unique fungal ASVs, respectively) (Figure 5b), underscoring the highly specific symbiotic relationships corals maintain with certain fungi and the distinct ecological groups they form [81,82,83]. PCoA analysis further corroborates the significant differences in fungal communities among the corals, emphasizing the specificity and stability of coral–fungal associations.

In addition, through functional predictions using the FUNGuild database, significant heterogeneity is observed in the distribution of fungal functional groups among different coral species, unveiling the diversity and intricate ecological functions of fungal communities within coral ecosystems. These findings not only affirm the crucial role of coral–fungi interactions and their synergistic impact on the ecosystem but also identify key interaction patterns between corals and fungi. For instance, undefined saprotrophic fungi in coral *Dg* might serve as “calcification helpers”, potentially utilizing carbonates in coral skeletons to aid in calcification and enhance coral resilience [84,85]. Endophytes in corals *Ck* and *St* may establish beneficial symbiotic relationships, offering nutrition or protection, thereby boosting coral growth and defense mechanisms [86,87]. Conversely, fungal parasites and wood-decaying fungi in coral *Fs* could act as “pathogenic adversaries” or “resource competitors”, impairing coral health and survival [88,89]. Additionally, the shared fungal genera between corals *Dg* and *Ck* hint at similarities in their environmental adaptability or symbiotic relations, possibly due to comparable microbial environments or resistance mechanisms [16,90]. The absence of certain fungal genera in corals *St* and *Ck*, classified based on their trophic types, might reflect the corals’ selective symbiotic preferences and these fungi’s potential roles in coral health and pathology [91,92]. Notably, the unique detection of Ectomycorrhizal fungi in coral *Dg* suggests specialized symbiotic relationships critical for nutrient cycling and environmental adaptation [93,94]. However, these functional predictions, derived from FUNGuild database assignments, should be considered preliminary. Further experimental studies are required to validate these ecological roles and better understand the functional contributions of fungal communities to coral health and resilience.

## 5. Conclusions

In conclusion, this study provides valuable insights into the fungal communities inhabiting four species of corals from the South China Sea, revealing their complex diversity and the potential implications for coral health. The finding that 10 fungal genera are firstly reported for corals in this study underscores the likelihood of underestimating fungal diversity and highlights the need for further exploration into the functions and adaptive mechanisms of these fungi. The distinct fungal communities, marked heterogeneity in functional groups, and identified interaction patterns underscore the intricate symbiotic relationships between corals and fungi. These insights offer novel perspectives and scientific evidence essential for coral conservation and management, advocating for a deeper exploration of coral microbial composition and function.

## Figures and Tables

**Table 1:** Diversity indices of the Shannon, Simpson, Richness, Chao1 and Ace indices.

Sample	Shannon	Simpson	Richness	Chao1	Ace
*Ck*1	1.74	0.74	44	44	44.48
*Ck*2	1.23	0.48	45	45	45.31
*Ck*3	0.69	0.24	25	25	25
Mean of *Ck*	1.22	0.49	38	38	38.26
*Fs*1	0.89	0.41	24	24	24.44
*Fs*2	0.55	0.24	15	15	16.08
*Fs*3	0.65	0.34	14	14	14
Mean of *Fs*	0.70	0.33	17.67	17.67	18.17
*St*1	1.51	0.60	58	58	58
*St*2	1.24	0.59	37	37	37
*St*3	1.05	0.49	37	37	37.44
Mean of *St*	1.26	0.56	44	44	44.15
*Dg*1	2.68	0.82	208	208.38	209.02
*Dg*2	1.79	0.65	128	128.13	128.442
*Dg*3	2.06	0.73	103	103.75	103.65
Mean of *Dg*	2.18	0.73	146.33	146.75	147.038

The number of sequences in each sample was standardized to 22,895 by the draw leveling process. The coral species analyzed included *Dichotella gemmacea* (*Dg*), *Cladiella krempfi* (*Ck*), *Sarcophyton tortuosum* (*St*), and *Favia speciosa* (*Fs*). Shannon and Simpson indices measure species diversity and evenness, while Richness, Chao1, and Ace indices estimate species richness.

## Data Availability

Data are contained within the article and Supplementary Materials.

## Supplementary Materials

The following supporting information can be downloaded at: https://www.mdpi.com/article/10.3390/jof10070452/s1.
